# Vaping and smokeless tobacco control in South Asia: A policy review

**DOI:** 10.1016/j.amsu.2022.104285

**Published:** 2022-08-01

**Authors:** Nishwa Azeem, Zouina Sarfraz, Azza Sarfraz, Namrata Hange, Muzna Sarfraz, Ivan Cherrez-Ojeda

**Affiliations:** a Lahore General Hospital, Lahore, Pakistan; b Fatima Jinnah Medical University, Lahore, Pakistan; c Aga Khan University, Karachi, Pakistan; d Eurasian Cancer Research Council, Mumbai, India; e King Edward Medical University, Lahore, Pakistan; f Universidad Espíritu Santo, Samborondón, Ecuador, Respiralab Research Center, Guayaquil, Ecuador

**Keywords:** Tobacco, Smoking, E-cigarette, ENDS, South Asia, Policy

## Abstract

High prevalence of tobacco use is seen in low- and middle-income countries (LMIC). In the recent years, tobacco users have switched to alternatives falling under the framework of electronic nicotine delivery systems (ENDS). This review provides an overview of tobacco control-related policies in Bangladesh, India, and Pakistan, and suggested recommendations to bridge this gap to address Tobacco-free Nations. This paper's findings are relevant for developing countries worldwide that have a high tobacco-related health burden, a complex landscape of use, and inadequate resources to offer tobacco cessation and smokeless tobacco control.

## Highlights

• Tobacco use and electronic nicotine delivery systems are peaking in LMIC.
• This review overviews tobacco-control policies in Bangladesh, India and Pakistan.
• Recommendations are listed to bridge the gap to address tobacco-free nations.

## 1 Introduction

There is a massive rise in the burden of tobacco use in low- and middle-income countries (LMICs), brought about due to misinformation and adverse marketing practices [1]. The World Health Organization (WHO) affirms that tobacco usage is the foremost cause of avertable deaths globally [1]. South Asia, is the largest area worldwide for the manufacturing and consumption of tobacco products, where more than 50% of the population is below the poverty line [1]. In Southeast Asia, tobacco is used in varied forms, including cigarettes or bidis (dried tobacco rolled in paper or leaf), smokeless tobacco (SLT) such as chewing khaini (tobacco with slaked lime and aromatic spices), *surti* (dried tobacco leaves for chewing), or *paan* masala (tobacco with aromatic spices), sucking gutkha (mixture of tobacco and molasses available in small sachets), applying *gul* or *gudaku* as dentifrice, and inhaling *nas* and *naswar* (nasal inhalation of tobacco powder) [2].

Smoking cessation is a viable method to halt the adverse effects of smoking [3]. While methods are employed for this purpose, smoking cessation pills available for users reportedly are potential carcinogens [4]. In recent years, previous tobacco users or novel users have considered switching to alternatives such as vaporizers, vape pens, e-pens, e-hookahs, e-pipes, and e-cigars, all of which are classified as electronic nicotine delivery systems (ENDS) [5]. ENDS are noncombustible tobacco products that are typically rechargeable through USB ports [5,6]. E-cigarettes vaporize “e-liquid” with each inhalation puff in a heating chamber, exposing the user to nicotine blended with a base (usually propylene glycol), concentrating flavorings along with chemicals predisposing the person to addiction and related adverse effects [7]. The e-liquid comes in different flavors and vastly different concentrations of nicotine depending on the brand and flavor choice [7]. Data suggests that the characteristics of ENDS products, including flavorings significantly affect the inhalation toxicity of aerosol generated from them [7].

ENDS has now become an ever-expanding market with the introduction of new products, ingredients, and devices. Scholarly venues and policy analysts must keep updated and understand the effects of these products on human health. There is a paucity of literature addressing the behavioral use of e-cigarettes and thereby a lack of conclusive results on the holistic adverse impact of conventional cigarette use concerning its behavioral use. This review collates evidence for use by public health bodies, governmental decision-makers, academicians, and others to weigh policies and population guidelines, which are reflective of i) the deleterious effects of vaping and smokeless tobacco, and ii) the trend of use in populations based on the same principles of WHO Framework Convention on Tobacco Control (FCTC) signed by South Asian countries. This review provides an overview of tobacco control-related policies in Bangladesh, India, and Pakistan, and suggested recommendations to bridge this gap to address Tobacco-free Nations.

## 2 Epidemiology

### 2.1 Prevalence of vaping and smokeless tobacco users

Globally, tobacco use among youth and adults is increasing in epidemic proportions [8]. A 2015 survey spanning 121 countries finds that 351.9 million smokeless tobacco (SLT) in some form and over 90% of these users live in South Asia [9]. With nearly 29% of adults using tobacco, India is the second dominant end-user of tobacco products after China accommodating 11% of the world's adult cigarette smokers [10,11]. Exclusively, 35% of Bangladeshi adults use tobacco, accounting for 37.8 million people, while 19.1% percent of Pakistani adults use tobacco in any form. Detailed classifications as given below in Table 1. Around 5% of the Pakistani youth use ENDS. India banned e-cigarettes and vaping in 2019, however, from 2014 to 2019, the sales volume increased from 1.6 to 3.3 million (a double increase). Bangladesh planned to ban e-cigarettes hence there are no proper statistics on how many people own and use ENDS in the country.

**Table 1:** Country-based specifications of tobacco, smokeless tobacco, and ENDS use [[12], [13], [14], [15]].

Country	Tobacco	Smokeless Tobacco	Total	Vaping/E-Cigarettes
India	99.5 million (10.7%)	199.4 million (21.4%)	266.8 million (28.6%)	268,000 0.02%
Pakistan	23.9 million (19.1%)	15.6 million (12.4%)	9.6 million (7.7%)	6.2%
Bangladesh	19.2 million (18.2%)	22 million (20.6%)	37.8 million (35.3%)	0.2%

Consumption of SLT among Pakistani females is reportedly much lower than the rate observed among Indian women (18.4%) and Bangladeshi (27.9%) females. There have been evident disparities among urban and rural populations considering India and Bangladesh, whereby there is a higher prevalence of chewing tobacco in rural areas; rural female tobacco usage is close to double the urban rate. The most commonly used oral smokeless tobacco product is *Zarda* (14.5%) in Bangladesh, *Khaini* (11.2%) in India, and *Naswar* (5.1%) in Pakistan. Most tobacco users are 25 years or older, with low literacy, low wealth index, and with greater exposure to marketing related to tobacco products [16,17]. E-cigarette users still constitute small proportions in India and Bangladesh; predominantly tobacco smokers are at a larger prevalence compared to SLT users with a majority transitioning to SLIT to quit or reduce tobacco use. There are substantial concerns about e-cigarettes usage in the Pakistani population as well considering increased popularity and usage in the young population.

### 2.2 Lung and oral cancer prevalence

According to the Global Burden of Disease Study, in 2019, tobacco caused the death of about 157,862 people, accounting for 19% of total deaths. Tobacco kills more than 1 million people every year in India which accounts for 9.5% of all deaths. Population-based cancer registries (PBCRs) from India have reported 370,000 cases of Tobacco-related cancer which accounts for 27.1% of India's total cancer burden [18,19]. Tobacco is the largest silent killer in Pakistan as 160,189 people die every year out of 23.9 million adult tobacco users. An overview is provided in Table 2.

**Table 2:** Statistical Overview of Tobacco-related deaths, lung and oral cancer prevalence [20].

Countries	Tobacco-related deaths	Lung cancer Incident cases	Death due to Lung cancer	Oral cancer Incidence	Deaths due to Oral cancer
**Bangladesh**	126,000 (of 164.7 million)	12,999 (8.3%)	12,003 (11%)	13,985 (8.9%)	8,137 (7.5%)
**India**	1.35 million (of 1.38 billion)	72,510 (5.5%)	66,279 (7.8%)	135,929 (10.3%)	75,290 (8.8%)
**Pakistan**	163,600 (of 220.9 million)	10,538 (5.9%)	9,288 (7.9%)	16,959 (9.5%)	10,617 (9.1%)

A recent study reports compendious monetary estimates of tobacco use on health and implications for tobacco control in Bangladesh. It finds that 125,718 tobacco-related diseases occur each year in Bangladesh, causing 13.5% of deaths [21]. India loses 1% of its GDP to diseases and early deaths from tobacco use. Total monetary expenditure secondary to all diseases and deaths from tobacco usage in persons 35 years or older amounts to INR 1773.4 billion ($27.5 billion), in 2017–2018; of which 78% are indirect costs [22]. For Bangladesh, 305.6 billion Bangladeshi taka (BDT) ($3.61 billion) is total economic expenditure with 72% in indirect costs of BDT221.7 billion ($2.6 billion) [21]. For the Pakistani Population, total financial costs attributable to all smoking-related diseases and deaths in 2019 account for PKR 615.07 billion ($3.3 billion). Total smoking-attributable costs account for 1.6% of the GDP while smoking-attributable costs of cancer, cardiovascular and respiratory diseases account for 1.15% of the GDP. Total revenue collected from the tobacco industry is worth only 20% of the total cost of smoking [23].

## 3 Policy comparisons

### 3.1 Policy on public use and advertisements

In indoor workplaces, public places, government facilities, hospitals, non-residential/residential healthcare facilities, pre-schools, schools, and playgrounds, individuals are prohibited from smoking by law. Violation of restrictions on smoking is subject to an infinitesimal penalty in these countries. In all three countries, the purchase of tobacco products is legal. The sale of tobacco products is restricted to the population below the age of 18 in all three countries. The law and legislation prohibit the trading of tobacco products within 100 m of academic or healthcare facilities for Bangladesh and India while Pakistan law specifies a boundary of 50 m.

Though regulated forms of advertising and promotional strategies are quite stringent in these three countries, however, internet tobacco product sales are permitted [24]. Purchase of tobacco products via coin-operated vending machines is legally not allowed in India and Bangladesh while still permitted in Pakistan. Reverse brand stretching (i.e., trademark diversification) includes the use of non-tobacco branding on tobacco products. Brand sharing includes using established tobacco brand elements or logos for non-tobacco products or services. Both are still permitted in Pakistan and Bangladesh, while legally banned in India. Brand marketing inclusive of characteristic words, badges, designs, images, emblems, trademarks, sounds, or colors to endorse tobacco products in entertainment locales, wholesale outlets, on automobile vehicles, and other physical structures, appliances is currently legalized in Pakistan. Legislation addressing the packaging and labeling of tobacco products in Bangladesh requires rotating pictographic health notifications to cover at least 50% of the prominent exhibition areas of all tobacco products. While for India, health admonition logos and Indian health warning labels are inclusive of photographic content, covering 85% of the front and rear packaging, parallel to the top edge; and annually rotating. The Ministry of Health - Pakistan has made a public warning occupying 60% of pictographic scripts to be placed on all cigarette packs in Urdu on the front and in English on the back.

The ‘Prohibition of Electronic Cigarettes Act' in India has addressed manufacturing, production, trading inclusive of import and export, transportation, sale, marketing, distribution, storage, and endorsement, despite no clear indication for personal use. On the contrary, no restrictions have been imposed on the retail sale, promotion, advertising, sponsorship, packaging, and labeling of e-cigarettes for Bangladesh and Pakistan. However, the Health Ministry ofBangladesh planned to enforce a ban on the manufacture, import, and trading of electronic cigarettes along with vaping tobacco to avert ill effects on health. More information is provided in Table 3 pertaining to policy components as applicable to South Asia.

**Table 3:** Policy components of manufacturing, distribution, and personal use in south Asia.

Policy Component	Afghanistan [25]	Bangladesh [26]	Bhutan [27]	India [28,29]	Maldives [30]	Nepal [31]	Pakistan [32]	Sri Lanka [33,34]
**Sales of E-Cigarettes**	Allowed	Allowed	Banned	Banned	Allowed	Banned	Allowed	Banned
**Use in Indoor, Public Places, Workplaces, and Public Transport**	Allowed	Allowed	Banned	No law (allowed by default)	Banned	Banned	Allowed	Allowed (No law)
**Advertising, Promotion, and Sponsorship**	Allowed	Allowed	Banned	Banned	Banned	Banned	Allowed	Allowed (No law)
**Point of sale product display**	Allowed (No law)	Allowed	N/A	N/A	Not Allowed	N/A	No Law	N/A
**Health Claims in advertising (modified risk claim)**	Allowed (No law)	Allowed	Not Allowed	N/A	N/A	N/A	Allowed	Allowed (No law)
**Sales of E-Cigarettes via the internet**	Allowed (No law)	Allowed	Allowed to import for personal use	Banned	Allowed (with limitations)	N/A	Allowed	N/A
**Favors**	Allowed	Allowed	N/A	N/A	No Law	N/A	Allowed	N/A
**Special Ingredients/additives**	No Law	N/A	N/A	N/A	No Law	N/A	N/A	N/A
**Health Warning on Product Packaging**	Not Required	N/A	N/A	N/A	Required	N/A	Not Required	N/A
**Other product packaging and labeling requirements**	Not Required	N/A	N/A	N/A	Required	N/A	Not Required	N/A
**Maximum Nicotine Concentration**	Not Required	Not Required	N/A	N/A	No Law	N/A	Not Required	N/A
**Device Requirements**	No Law	Not Required	N/A	N/A	No Law	N/A	No Law	N/A
**Manufacturer/importer disclosures and/or notification requirements**	No Law	No Law	Required for imported products Tobacco Control Law 2004 and 2010	The Prohibition of Electronic Cigarettes (Production, Manufacture, Import, Export, Transport, Sale, Distribution, Storage and Advertisement) Bill, 2019	Required Act No.: 15/2010 (Tobacco Control Act)	N/A The Tobacco Product Control and Regulatory Directive, 2014	No Law	Prohibited Tobacco Products Regulations, No.1 of 2016

### 3.2 Policy on taxes and subsidy

India being the second-largest population of adult smokers in the world, banned the sale of e-cigarettes in October 2019. Globally, it has been cautioned that there is a “vaping epidemic” occurring among young people. However, in Pakistan, the purchase and use of E-Cigarettes are allowed. According to the WHO report addressing the global tobacco epidemic in 2019, compliance with smoking-free policies is low in Pakistan (score 3) while India and Bangladesh are in the average range (6–7) [35]. A brief overview of taxation across Bangladesh, India, and Pakistan for cigarette prices may be seen in Table 4.

**Table 4:** Taxation and excise taxes as a percentage of cigarette prices [1,36].

	WHO	Bangladesh	India	Pakistan
Taxation a2019 (MPOWER)	–	71%	54%	56.4%
Excise tax as a percentage of cigarette price	70–75% Recommended	61%	52%	41.83%

a Variable for other tobacco products.

## 4 Recommendations

As with WHO's Framework Convention on Tobacco Control (FCTC) which was first negotiated and entered into force in 2005, it is essential to address the many shortcomings of current treaties in curbing the SLT and vaping uprising in South Asia. It is recommended to begin by collecting data based on the global youth tobacco survey (GYTS), which is supported by the WHO. More data collation across different cities in South Asian countries will specify socioeconomic differences among users. This will improve policy-making decisions. More and more SLT products find leeway in advertising (i.e., sponsoring races, and programs), hence decision-makers need to keep updated on the numerous marketing strategies in the emerging SLT and vaping economy.

**Fig. 1:**
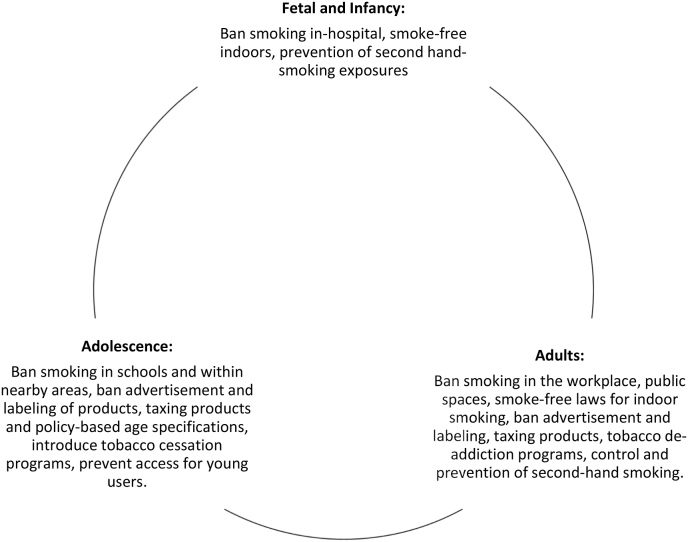
Policy ramifications for a smoke-free world: Life course approach.

Visible and aggressive anti-tobacco campaigns may target users based on a life-course approach (Fig. 1). There is a need to design this intervention based on newly collated evidence that requires tailoring either to GYTS or other credible data collection tools. The behavioral components of interventions may be added before the individual turns 18 years of age to improve compliance. This can be proceeded by reviewing school-based activities and strictly enforcing legislation on access to tobacco in under-18 age groups. On the other hand, tobacco control strategies focused on ages 18 and older must address those with lower-level of education, of low socioeconomic status, and those that are prone to marketing [17]. All three countries are suggested to impose laws addressing the regulation of the contents of tobacco-based cigarettes with mandatory disclosure by manufacturers and importers to government authorities of information on the contents and emissions of their products.

Elevating taxes on tobacco is a documented, cost-efficient approach to tobacco control and minimizing the negative health and economic impacts of tobacco consumption. A viable method to do this is through a uniform specific excise tax that comprises at least 75% of the retail price, as recommended by WHO. It would also help enforce existing laws and legislations through financial penalties for first-time and repeat offenders.

## 5 Conclusion

Over the last few decades, South Asian countries such as India, Pakistan, and Bangladesh have tried to work effectively to address tobacco control. However, with the looming threat of ENDS and SLT uptake, said countries ought to apply proven tobacco control tools that work for citizens’ holistic health addressing all levels among different age groups. This paper's findings are relevant for developing countries worldwide that have a high tobacco-related health burden, a complex landscape of use, and inadequate resources to offer tobacco cessation and SLT control. With India having implemented a ban on SLT and vaping use, other countries may slowly follow suit. It is essential to account for tobacco control programs and the inter-links these have with SLT and vaping since public health authorities are under-equipped given the lack of research into trends.

## Ethical approval

None required.

## Source of funding

None.

## Author contribution

All authors contributed equally to all aspects of this study. Nishwa Azeem and Zouina Sarfraz are co first authors.

## Trail registry number

Not required.

## Guarantor

Zouina Sarfraz is the guarantor of this research.

## Declaration of competing interest

None.
